# Management of multiple magnetic foreign body ingestion in pediatric patients

**DOI:** 10.1186/s12887-022-03501-0

**Published:** 2022-07-26

**Authors:** Yi Jin, Zhigang Gao, Yuebin Zhang, Duote Cai, Di Hu, Shuhao Zhang, Jianhua Mao

**Affiliations:** 1 Department of General Surgery, Children’s Hospital, Zhejiang University School of Medicine, National Clinical Research Center for Child Health, Hangzhou, 310051 Zhejiang Province China; 2 The Children’s Hospital, Zhejiang University School of Medicine, National Clinical Research Center for Child Health, Hangzhou, 310051 Zhejiang Province China

**Keywords:** Multiple magnetic foreign body, Children, Management, Gastroscope, Surgery

## Abstract

**Background:**

Multiple magnetic foreign body ingestion in children is increasingly common and can cause serious injury. The present study aimed to analyze the clinical features of such cases and summarize treatment experiences.

**Methods:**

A retrospective survey of 91 patients in the Children’s Hospital, Zhejiang University School of Medicine with magnetic foreign body ingestion from October 2018 to October 2021 was performed, the data were collected including the clinical information of the patients, treatment details, and prognosis.

**Results:**

Twenty-two (24.2%) patients were conservatively treated, with the foreign bodies discharged through the anus, 31 (34.1%) underwent laparoscopic surgery, including 18 cases converting from laparoscopic surgery to laparotomy, and 38 (41.8%) underwent laparotomy. In 13 (14.3%) patients, the foreign bodies were partially removed by gastroscope. The remaining foreign bodies were removed by laparoscopy in six patients, including three cases converting from laparoscopy to laparotomy, by laparotomy in four patients, and by conservative treatment in three patients.

**Conclusions:**

Multiple magnetic foreign body ingestion can cause significant harm to patients and different clinical techniques must be used for patients in different situations to reduce the harm to children.

## Introduction

Multiple magnetic foreign body (MMFB) ingestion in children has increased rapidly in recent years [1]. Unlike other ingested foreign bodies, MMFB can cause serious consequences such as intestinal necrosis, ileus, and perforation by being able to connect to one another across the loops of the bowel [2]. Thus, it is very important for pediatricians to understand the harm of the MMFB and know how to deal with this situation. In the present study, a retrospective analysis was performed on 91 patients who ingested MMFB, and the management of this disease was summarized to provide information for other clinicians.

## Materials and methods

### Patients and clinical data

The clinical data of 91 patients with MMFB ingestion who were admitted to the Department of General Surgery, Children’s Hospital, Zhejiang University School of Medicine from October 2018 to October 2021 was collected. Abdominal x-ray was used to determine the diagnosis and the clinical characteristics, including sex, age, pre-operative symptoms, treatment methods and prognosis were described and analyzed. This study was approved by the Ethical Committee of The Children’s Hospital, Zhejiang University School of Medicine (No. 2020-IRB-120) and all methods were performed in accordance with the relevant guidelines and regulations.

### Statistical analysis

Statistical analysis was performed with SPSS 24.0. Pearson’s chi-square and Fisher’s exact tests were used for categorical variables and the frequencies were reported as a percentage of the group of origin. The Mann–Whitney U test was utilized for continuous variables and frequency of continuous variables was reported as the median and interquartile ranges (IQR). *P*-Values < 0.05 were considered statistically significant. All *P*-values reported were two-tailed.

## Results

A total of 22 patients (14 males and 8 females) received conservative treatment. The median age of the patients was 61 months (IQR: 40.75–93 months) with a median weight of 18.75 kg (IQR: 14.75–25.63 kg). All patients were asymptomatic and the abdominal x-ray showed a meaningful change in the position of the foreign body, among them, 13 patients (59%) could tell the doctor with great certainty that the foreign bodies were swallowed at one time. The foreign bodies were discharged through the anus without medical intervention.

A total of 69 patients consisting of 52 males and 17 females received surgical treatment. The median age of those patients was 43 months (IQR: 21–67 months) with a median weight of 15.5 kg (IQR: 12.25–20 kg). The most common symptom was abdominal pain and vomiting. Thirty-eight cases underwent laparotomy and 31 cases underwent laparoscopy, with 18 cases converted to laparotomy. The small intestine was the most frequent site for magnet adsorption, the main surgical approach was perforation repair. If the perforation could not be repaired, intestinal resection and anastomosis were performed. For the laparoscopic surgery group, the diseased bowel was exteriorized through an enlarged umbilical incision, followed by foreign body removal and repair, so the patients suffered less trauma and had a quicker recovery compared to the laparotomy group. The conversion of 18 children from laparoscopy to laparotomy was due to obvious abdominal distension or difficulty in exteriorizing the diseased bowel through the umbilicus incision (Table 1).

**Table 1:** Clinical parameters of the patients

Group	n	Gender (male/ female)	Age [months, M(P25-P75)]	Weight [Kg, M(P25-P75)]	Symptom(Symptomatic/asymptomatic)	Ingestion time( Definite/undefinite)	Foreign bodies number [M(P25-P75)]	Complications
conservative treatment group	22	14/8	61 (40.75–93)	18.75 (14.75–25.63)	0/22	19/3	3.5 (2–7)	none
surgery group								
laparotomy	38	28/10	35.5 (19.5–53.5)	14.48 ( 11.35–17.65)	27/11	14/24	8 (4.75–12)	3
laparoscope	31	24/7	49 (25–75)	16.5 (13.3–23)	16/15	17/14	5 (3–15)	1

The foreign bodies of 13 cases were partially removed by gastroscope and the remaining foreign bodies were excreted without further medical interventions in three cases, removed by laparoscopic surgery in six cases, including three cases converted from laparoscopy to laparotomy, and removed by laparotomy in four cases (Table 2).

**Table 2:** Clinical parameters of the patients whose foreign bodies were partial removed by gastroscope

Patient	sex	age	Weight(KG)	Ingestion time(days)	foreign bodies number	Symptoms	foreign bodies number (removed / residual)	Subsequent treatment	complications
1	Male	1Y6M	12	undefinite	19	vomiting	12/7	laparotomy	ileus
2	Male	4Y3M	15.2	14	18	abdominal pain and vomiting	6/2	laparotomy	none
3	Male	5Y2M	20	2	8	abdominal pain and vomiting	4/4	laparotomy	none
4	Male	2Y11M	13.5	undefinite	12	abdominal pain and vomiting	10/2	laparotomy	none
5	Male	4Y3M	15	21	3	none	1/2	Conversion from laparoscope to laparotomy	none
6	Femalee	3Y3M	11	undefinite	18	abdominal pain and vomiting	16/2	Conversion from laparoscope to laparotomy	none
7	Male	3Y7M	16	undefinite	22	none	7/15	Conversion from laparoscope to laparotomy	none
8	Male	2Y4M	15	undefinite	20	none	18/2	laparoscope	none
9	Male	4Y1M	20	1	31	none	26/5	laparoscope	none
10	Male	1Y6M	13	30	4	none	3/1	laparoscope	none
11	Male	3Y10M	18.5	6	2	none	0/2^a^	conservative	none
12	Male	9Y	39.6	7	75	none	58/17	conservative	none
13	Male	2Y1M	12.5	undefinite	7	none	5/2	conservative	none

^a^For this patient, the foreign body in the stomach was seen during gastroscopy, when grasping, the foreign body slipped to the depth of serosa and could not be found again. The patient had no symptoms, so he received conservative treatment. After 2 days, the foreign bodies were discharged through the anus

All patients were examined by x-ray to confirmed that there was no foreign body residue in the body.

## Discussion

Multiple magnetic foreign body (MMFB) ingestion in children has gradually become a global problem nowadays [1, 3]and require special attention. When multiple magnetic foreign bodies are ingested, they can attract each other across the bowel walls, leading to acute complications, including intestinal obstruction, perforation and peritonitis or death [4].

For the management of this disease, several algorithms have been published [5], but due to the complexity of the situation, there is currently no clear consensus, especially for the timing of endoscopy and the treatment of post-pyloric magnets [6, 7]. If the foreign bodies are in the esophagus or stomach, there is a clear consensus that gastroscope is the best method of removal [8], but if an x-ray shows that foreign bodies are located in the middle or lower abdomen (beyond the stomach), some researchers recommend surgical management [9], while the North American Society for Pediatric Gastroenterology, Hepatology, and Nutrition (NASPGHAN) recommends endoscopy if patients are asymptomatic [7]. In addition, NASPGHAN recommends that clinicians consult pediatric surgeons prior to endoscopic removal if ingestion is greater than 12 h prior to the time of procedure as ulceration and indentation of the mucosa may occur in less than eight hours [10]. However, the exact time of ingestion is often unknown.

**Fig. 1:**
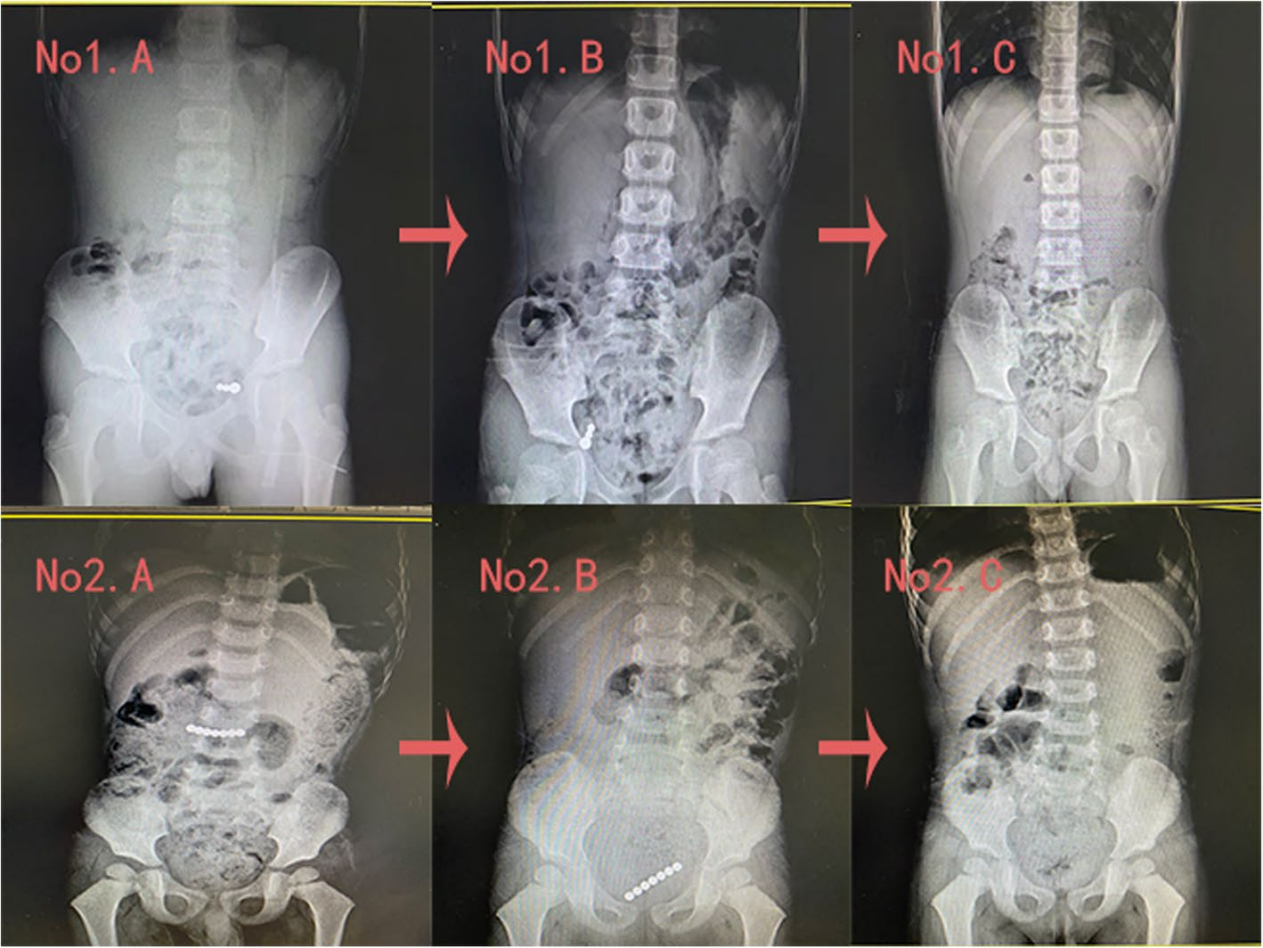
Patient 1 was asymptomatic and informed the doctor that the foreign bodies were swallowed at the same time. Serial x-rays showed that the foreign bodies moved and were excreted from the body. Patient 2 was asymptomatic and the doctor did not know if the foreign bodies were ingested at the same time. Serial x-rays showed a notable change in the position of the foreign bodies and they were discharged through the anus without medical interventions

This study showed that the patient’s medical history is very important, especially for elder children. If the patient is asymptomatic and informed the doctor that the multiple foreign bodies were swallowed at the same time, the patient can receive conservative treatment with hospital admission for further monitoring and serial x-rays; most such foreign bodies will pass through the digestive tract without medical intervention (Fig. 1). However, if the magnetic foreign bodies remain in the same position on sequential abdominal x-rays, surgical treatment should be considered even if the patient has no symptoms, because for such patients, intestinal fistula may have been formed [11].

**Fig. 2:**
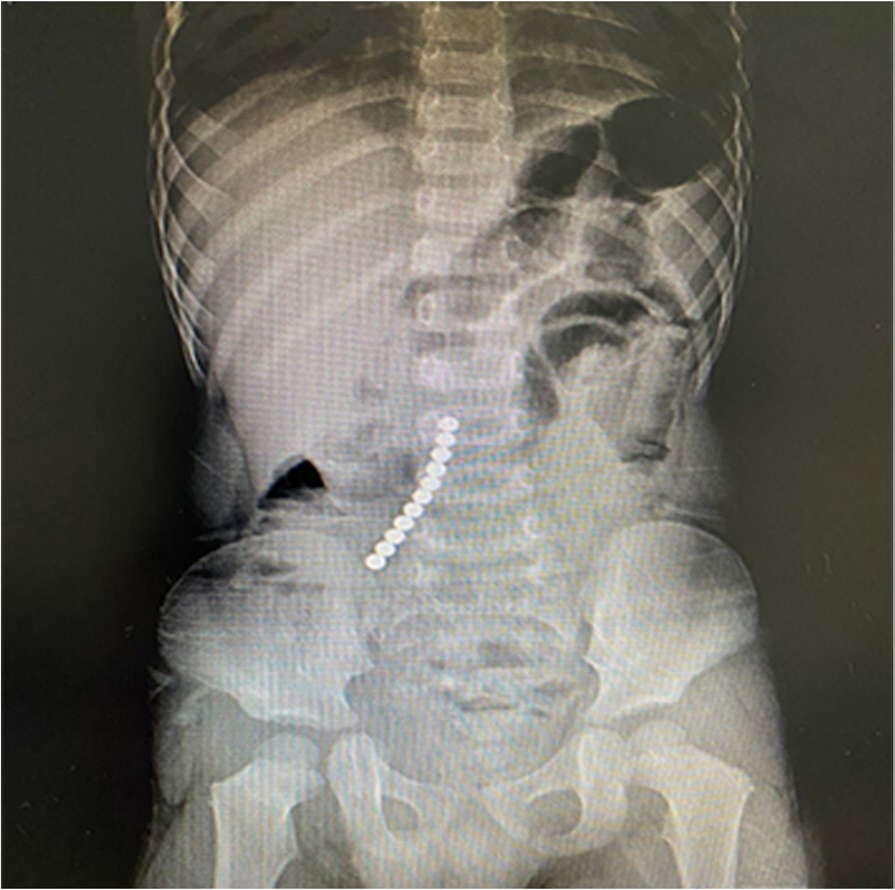
The x-ray showed that the foreign bodies were in the middle and lower abdomen. The patient had obvious symptoms of abdominal pain and vomiting. After laparotomy, it was found that the foreign bodies were in the stomach and small intestine, which attracted each other and caused injury

**Fig. 3:**
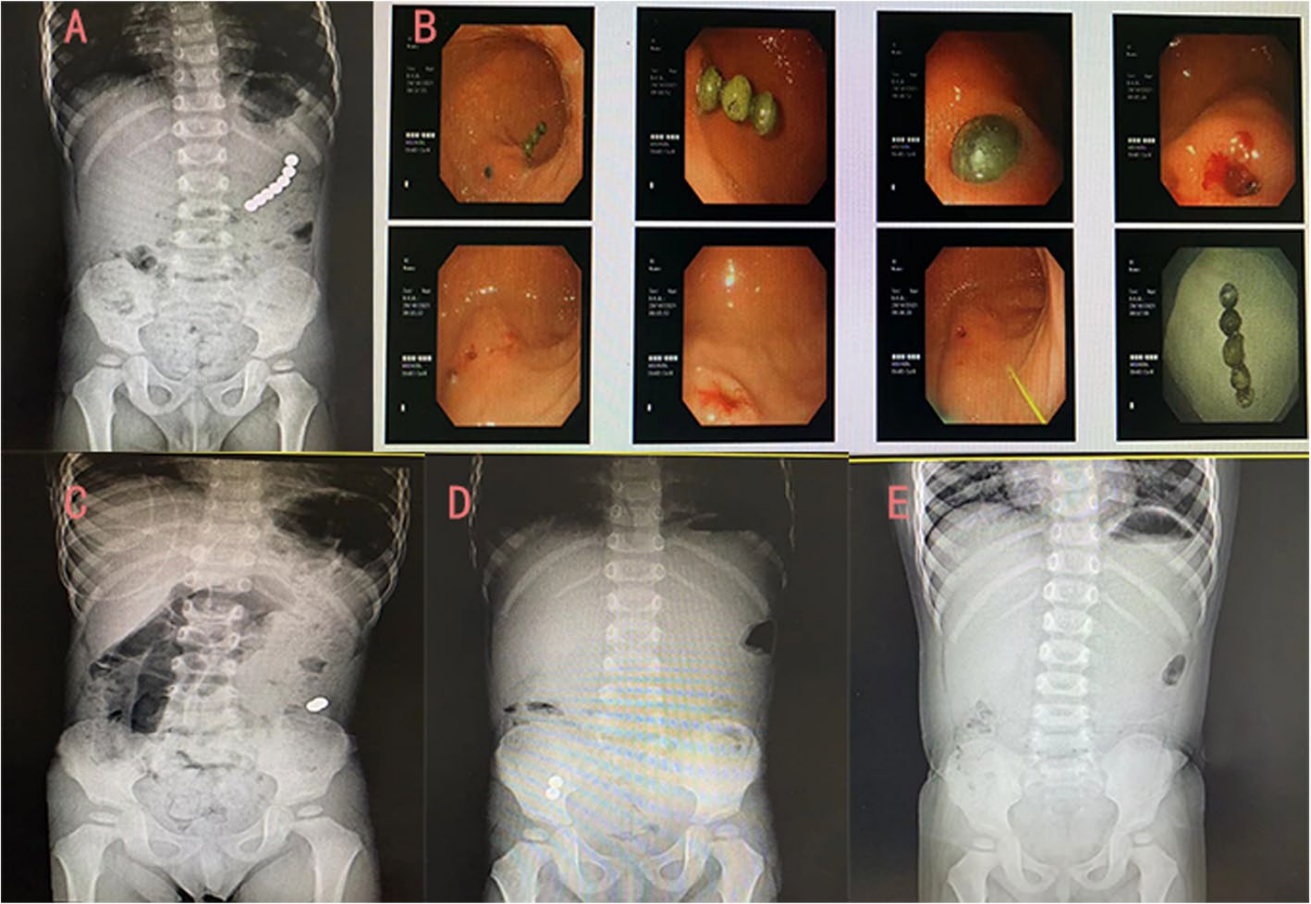
The x-ray showed that the foreign bodies were in the left upper abdomen, and thus, gastroscope was performed. It was found that there were five foreign bodies in the stomach. After being removed by gastroscope, an obvious depression was left on the gastric wall. The patient had no symptoms of pneumoperitoneum and peritonitis, so conservative treatment led to the two remaining foreign bodies being discharged through the anus

Gastroscope is really important for the treatment of this disease, no matter when the patient swallowed the foreign bodies. If the multiple magnetic foreign bodies are clearly diagnosed by plain abdominal x-ray, and their location is not in the pelvic cavity, gastroscopy should be considered first, because magnetic foreign bodies can attract each other, x-ray could not accurately show whether the foreign bodies are in the stomach (Fig. 2); if the magnetic foreign bodies are identified by gastroscope whether they are in the stomach, surgeons would know which kind of incision they can choose, this would not only facilitate the surgeon’s operation, but also reduce the length of the incision and the trauma to the patient; in addition, if the magnetic foreign bodies in the stomach are removed by gastroscope, and there is no sign of pneumoperitoneum or peritonitis for the patient, removal of the remaining foreign bodies can be delayed to observe if passage will occur via the anus without medical intervention (Fig. 3).

**Fig. 4:**
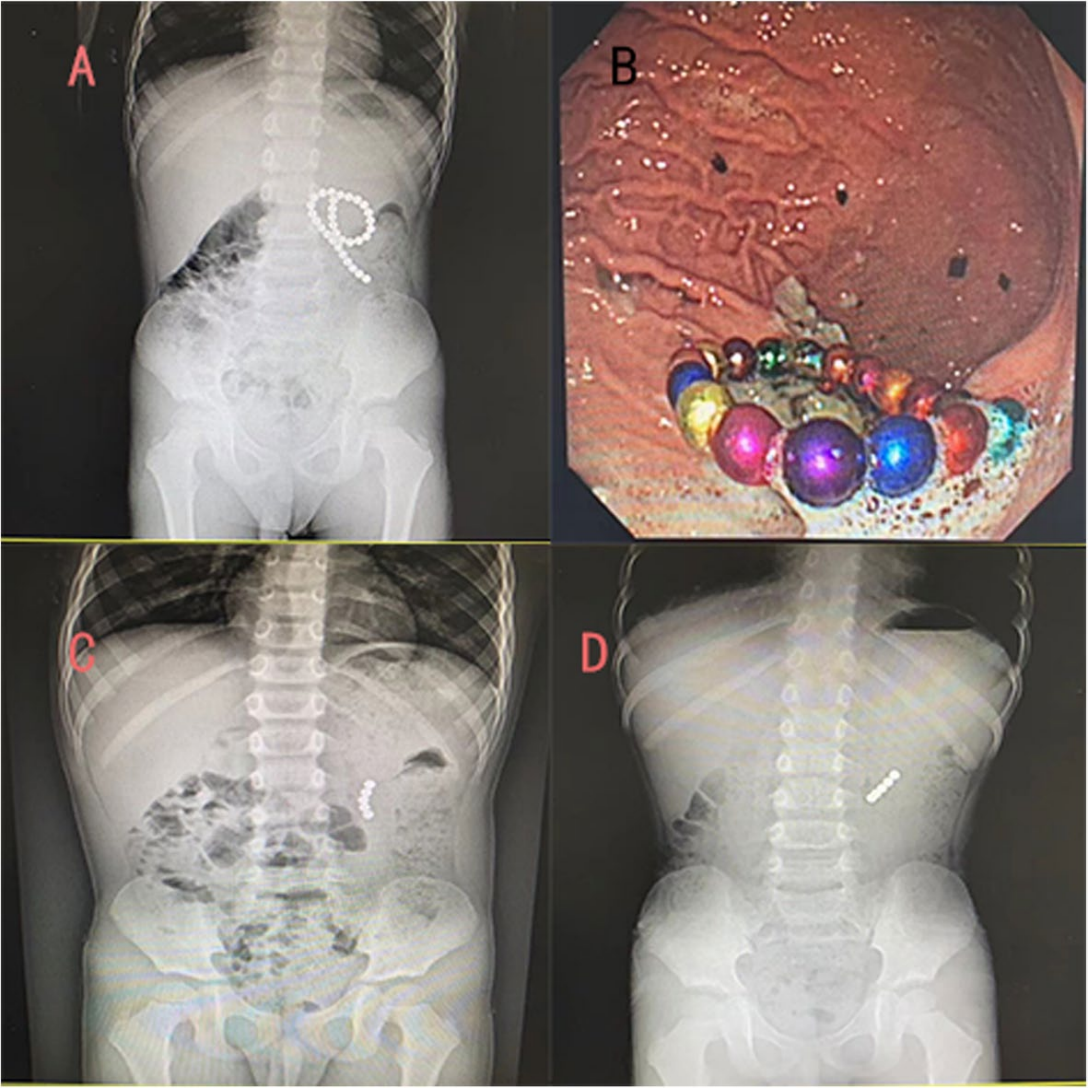
The x-ray showed that the foreign bodies were in the left upper abdomen, so gastroscope was performed. It was found that there were 26 foreign bodies in the stomach. After removal under gastroscope, the patient had no obvious abdominal symptoms, but continuous x-ray examination showed that the position of foreign bodies had no significant change. Considering the difficulty of self-discharge, the foreign body was surgically removed

If the patient still has symptoms such as vomiting, abdominal pain, or the remaining magnetic foreign bodies remain in the same position on sequential abdominal x-rays, surgical intervention is required (Fig. 4). The two types of surgical methods used are laparotomy and laparoscopy, some researchers recommended laparoscopic removal [4, 12], while some researchers believed that open surgery is the first choice [13], in our opinion, considering the minimally invasive of laparoscopic surgery, laparoscopy should be attempted first; when laparoscopic exploration find that only the small intestine is adsorbed by foreign bodies, the operator can expand the umbilical incision, pull the intestinal tube out of body, remove the foreign bodies and repair the intestine; in addition, if only a few intestinal tubes are sucked by foreign bodies, and there is no obvious intestinal necrosis and adhesion, surgeons can operate in the abdominal cavity to remove foreign bodies and repair intestinal tubes, when the situation is not suitable for laparoscopic operation, surgeons need to convert it to open surgery to avoid further complications. In the present study, there were 31 patients in the laparoscopic group, of which 18 (58%) were converted to open surgery. The reason for the high conversion rate was that magnetic foreign bodies often attracted each other across multiple digestive tracts. When magnets were partially located in the duodenum, colon, et. al, it was difficult to exteriorize the diseased bowel through the umbilicus incision. If magnets were forcibly removed by laparoscopy, celiac pollution and residual magnet beads may happen, also tiny perforations may be overlooked, resulting in serious postoperative complications.

The limitations of this study were that it is retrospective, descriptive, and had a small sample size of cases from a single pediatric surgery center. Future studies should be more extensive to produce more reliable results and produce reliable advice.

## Conclusion

Multiple magnetic foreign bodies can cause serious injuries to children, we call upon governments to introduce policies to ban the sale of such magnetic toys, or at least establish effective warning labels to keep children away from such toys, we also need to strengthen publicity to make the parents aware of the harm of magnetic toys. Gastroscope is important for the treatment of this disease, and when surgery is needed, considering laparoscopic exploration first but also preparing to switched to open surgery.

## Data Availability

The datasets used and/or analysed during the current study are available from the corresponding author on reasonable request.

## References

[1] Strickland M, Rosenfield D, Fecteau A (2014). Magnetic foreign body injuries: a large pediatric hospital experience. J Pediatr.

[2] Naji H, Isacson D, Svensson JF, Wester T (2012). Bowel injuries caused by ingestion of multiple magnets in children: a growing hazard. Pediatr Surg Int.

[3] Silverman JA, Brown JC, Willis MM, Ebel BE (2013). Increase in pediatric magnet-related foreign bodies requiring emergency care. Ann Emerg Med.

[4] Sola R, Rosenfeld EH, Yu YR, St Peter SD, Shah SR (2018). Magnet foreign body ingestion: rare occurrence but big consequences. J Pediatr Surg.

[5] Butterworth J, Feltis B (2007). Toy magnet ingestion in children: revising the algorithm. J Pediatr Surg.

[6] Vijaysadan V, Perez M, Kuo D (2006). Revisiting swallowed troubles: intestinal complications caused by two magnets–a case report, review and proposed revision to the algorithm for the management of foreign body ingestion. J Am Board Fam Med.

[7] Kramer RE, Lerner DG, Lin T, Manfredi M, Shah M, Stephen TC, Gibbons TE, Pall H, Sahn B, McOmber M (2015). Management of ingested foreign bodies in children: a clinical report of the NASPGHAN Endoscopy Committee. J Pediatr Gastroenterol Nutr.

[8] Geng C, Li X, Luo R, Cai L, Lei X, Wang C (2017). Endoscopic management of foreign bodies in the upper gastrointestinal tract: a retrospective study of 1294 cases. Scand J Gastroenterol.

[9] Lee HJ, Kim HS, Jeon J, Park SH, Lim SU, Jun CH, Park SY, Park CH, Choi SK, Rew JS (2016). Endoscopic foreign body removal in the upper gastrointestinal tract: risk factors predicting conversion to surgery. Surg Endosc.

[10] Hussain SZ, Bousvaros A, Gilger M, Mamula P, Gupta S, Kramer R, Noel RA (2012). Management of ingested magnets in children. J Pediatr Gastroenterol Nutr.

[11] Taher H, Azzam A, Khowailed O, Elseoudi M, Shaban M, Eltagy G (2019). A case report of an asymptomatic male child with multiple entero-enteric fistulae post multiple magnet ingestion. Int J Surg Case Rep.

[12] Huang YK, Hong SX, Tai IH, Hsieh KS (2021). Clinical characteristics of magnetic foreign body misingestion in children. Sci Rep.

[13] Huang X, Hu J, Xia Z, Lin X (2021). Multiple magnetic foreign body ingestion in pediatric patients: a single-center retrospective review. Pediatr Surg Int.

